# sTWEAK as Predictor of Stroke Recurrence in Ischemic Stroke Patients Treated With Reperfusion Therapies

**DOI:** 10.3389/fneur.2021.652867

**Published:** 2021-05-11

**Authors:** Pablo Hervella, María Pérez-Mato, Manuel Rodríguez-Yáñez, Iria López-Dequidt, José M. Pumar, Tomás Sobrino, Francisco Campos, José Castillo, Andrés da Silva-Candal, Ramón Iglesias-Rey

**Affiliations:** ^1^Clinical Neurosciences Research Laboratory (LINC), Health Research Institute of Santiago de Compostela (IDIS), Santiago de Compostela, Spain; ^2^Neuroscience and Cerebrovascular Research Laboratory, La Paz University Hospital, IdiPAZ, UAM, Madrid, Spain; ^3^Stroke Unit, Department of Neurology, Health Research Institute of Santiago de Compostela (IDIS), Hospital Clínico Universitario, Santiago de Compostela, Spain; ^4^Department of Neuroradiology, Hospital Clínico Universitario, Health Research Institute of Santiago de Compostela (IDIS), Santiago de Compostela, Spain

**Keywords:** MRI, prognosis, stroke prevention–primary & secondary, leukoaraiosis, stroke recurrence

## Abstract

**Aim:** The purpose of this study was to investigate clinical and neuroimaging factors associated with stroke recurrence in reperfused ischemic stroke patients, as well as the influence of specific biomarkers of inflammation and endothelial dysfunction.

**Methods:** We conducted a retrospective analysis on a prospectively registered database. Of the 875 patients eligible for this study (53.9% males; mean age 69.6 ± 11.8 years vs. 46.1% females; mean age 74.9 ± 12.6 years), 710 underwent systemic thrombolysis, 87 thrombectomy and in 78, systemic or intra-arterial thrombolysis together with thrombectomy was applied. Plasma levels of interleukin 6 (IL-6) and tumor necrosis factor alpha (TNFα) were analyzed as markers of inflammation, and soluble tumor necrosis factor-like inducer of apoptosis (sTWEAK) as an endothelial dysfunction marker. The main outcome variables of the study were the presence and severity of leukoaraiosis (LA) and stroke recurrence.

**Results:** The average follow-up time of the study was 25 ± 13 months, during which 127 patients (14.5%) showed stroke recurrence. The presence and severity of LA was more severe in the second stroke episode (Grade III of the Fazekas 28.3 vs. 52.8%; *p* < 0.0001). IL-6 levels at the first admission and before reperfusion treatment in patients with and without subsequent recurrence were similar (9.9 ± 10.4 vs. 9.1 ± 7.0 pg/mL, *p* = 0.439), but different for TNFα (14.7 ± 5.6 vs. 15.9 ± 5.7 pg/mL, *p* = 0.031) and sTWEAK (5,970.8 ± 4,330.4 vs. 8,660.7 ± 5,119.0 pg/mL, *p* < 0.0001). sTWEAK values ≥7,000 pg/mL determined in the first stroke were independently associated to recurrence (OR 2.79; CI 95%: 1.87–4.16, *p* < 0.0001).

**Conclusions:** The severity and the progression of LA are the main neuroimaging factors associated with stroke recurrence. Likewise, sTWEAK levels were independently associated to stroke recurrence, so further studies are necessary to investigate sTWEAK as a therapeutic target.

## Introduction

Since the implementation of the new approach to stroke as a neurological emergency, which has led to the progressive creation of Stroke Units and the development of new reperfusion therapies, short-term outcome has improved in developed countries (1–4) both in terms of mortality and functional outcome. The new guidelines, however, have focused mainly on patient care in the acute phase, but we have not seen many developments in post-hospital care, or secondary prevention, and some data suggest an increase in late disability in stroke patients (2).

A large part of early, medium and late morbimortality is associated with stroke recurrence, which affects 40% at 5 years and 50% at 10 years after the first cerebrovascular episode, both ischemic and hemorrhagic (5–9). The control of vascular risk factors, antiplatelet agents and statins has not significantly modified therapeutic strategy, although direct oral anticoagulant drugs have fundamentally demonstrated fewer hemorrhagic complications (10, 11).

The influence of reperfusion therapies in acute phase on stroke recurrence has not been well-established. It seems, however, that in patients undergoing mechanical thrombectomy early recurrence is lower, but in patients that receive intravenous thrombolysis medium and long-term recurrence is similar. In both cases, reperfused patients seem to have a better long-term progress as compared to non-reperfused patients (1, 12–14).

On the other hand, there is clinical evidence that moderate to severe leukoaraiosis ((LA) or white matter lesions) presence may be related with endothelial dysfunction and blood brain barrier (BBB) damage (15–18). LA presence is known to contribute to long-term functional decline, morbidity, and death in independent outpatients and in stroke patients (19). We have recently identified an endothelial dysfunction marker, the soluble tumor necrosis factor-like inducer of apoptosis (sTWEAK), as a possible biomarker independently associated with hemorrhagic transformation and poor functional outcome in patients with IS undergoing reperfusion therapies through the presence of LA (20). sTWEAK is constitutively expressed by monocytes, tumor cell lines, and endothelial cells. Via binding to fibroblast growth factor-inducible 14 [Fn14]), sTWEAK can function as an inflammatory cytokine. In this line, previous studies have shown that patients with IS had high sTWEAK levels. However, no correlation was found between sTWEAK and an ischemic area volume during acute stroke (21, 22).

At present, the primary goal of secondary prevention strategies after IS is to reduce the risk of recurrent stroke, and information on stroke recurrence and survival is useful to assess the effect of secondary prevention and risk factors for recurrence and death. In this scenario, it would be useful to identify biomarkers that could become therapeutic targets for developing future treatments or diagnostic indicators for stroke recurrence prevention; which would allow more accurate post-hospital follow-up/care, as this would lead to lower disability and mortality in medium and long-term outcome.

We hypothesized that elevated serum levels of sTWEAK might be involved in a higher frequency of stroke recurrence through the presence of LA. In the present study, we intend to investigate the possible relationship among sTWEAK—LA—stroke recurrence in reperfused IS patients; compare results with other inflammation biomarkers and evaluate the functional outcome at 3 months.

## Materials and Methods

### Patient Screening

For this study, we enrolled the stroke patients admitted to the Stroke Unit of the Hospital Clínico Universitario of Santiago de Compostela (Spain), who were prospectively registered in an approved data bank (BICHUS), and received reperfusion therapies (both intravenous and endovascular) during the acute phase. All patients were treated by expert neurologists according to national and international guidelines. Exclusion criteria for this analysis were: (1) latency time (from the onset of symptoms to hospital care) >4.5 h; (2) previous modified Rankin scale (mRS) >1; (3) history of chronic inflammatory diseases; (4) lack of at least two neuroimaging studies in the 1st week; (5) lost to follow-up patients (personal interview or telephone) at 3 months. The analysis of the data for this study was retrospective, using the period between September 2007 and September 2017.

For the estimation of stroke recurrence (ischemic stroke (IS) or intracerebral hemorrhage (ICH) patients) after the first ischemic stroke, the same database (BICHUS) was used in patients re-admitted to the same Stroke Unit. All the patients under care in Galicia (Spanish region on the northwest of the Iberian Peninsula) by the Servizo Galego de Saúde (SERGAS) are registered in a computer medical history (IANUS) that was used for patients who presented recurrence and who were seen by primary care doctors or other hospitals in the public network. Patients treated in private centers or outside Galicia were not registered and consequently excluded.

### Clinical Variables and Neuroimaging Studies

The registry includes demographic variables, vascular risk factors, time from stroke onset to reperfusion therapies, comorbidities and associated treatments, axillary temperature and blood pressure, blood count and coagulation test, and biochemical variables. The clinical picture was evaluated by certified neurologists using the National Institute of Health Stroke Scale (NIHSS) at admission, every 6 h during the 1st day, and every 24 h during hospitalization; modified Rankin Scale (mRS) was used to evaluate functional outcome at discharge and at 3 months. Effective reperfusion was defined as ≤ 8 points in the NIHSS during the first 24 h. Poor outcome was defined as mRS > 2 at 3 months. Stroke diagnosis was made using the TOAST classification (23).

In the first episode, Computed Tomography (CT) was performed in all patients and Magnetic Resonance Imaging (MRI) in selected patients at admission. Follow-up CT scan after fibrinolysis or thrombectomy was performed in all patients at 24 h, and CT at 48 h or at any time if neurological deterioration (increase ≥4 points in the NIHSS) was detected; and between the 4th and 7th day. The presence and severity of LA was assessed using Fazekas scale (24) with a total score of 0 to 6 (Fazecas I or Grade I, 1-2; Fazecas II or Grade II, 3-4; Fazecas III or Grade III, 5-6) by MRI/CT. Hemorrhagic transformation was defined according to ECASS II criteria (25). All neuroimaging tests were analyzed by a neuro-radiologist supervised by the same researcher (JMP). The neuroimaging study was completed in 786 (89.8%) patients. In the recurrence episode, in 94 (74.0%) patients only one CT was performed at admission and in 68 (53.5%) patients a further study was performed between the 4th−7th day.

### Biomarkers

We used plasma levels of interleukin 6 (IL-6) and tumor necrosis factor alpha (TNFα) as markers of inflammation, and soluble tumor necrosis factor-like inducer of apoptosis (sTWEAK) as marker of endothelial dysfunction (26, 27). The blood sample to measure biomarkers was collected before the administration of the reperfusion treatment in the first stroke, and in the case of a recurrent stroke, in the 1st h following admission to the Stroke Unit of the Hospital Clínico Universitario of Santiago de Compostela. In the first episode, IL-6 measurements were performed in 843 patients (96.3%), TNFα in 828 (94.6%) and sTWEAK in 869 (99.3%). In the recurrences, the percentage of patients with a sample to measure biomarkers was lower (IL-6 71.6%; TNFα 56.7%; and sTWEAK 67.7%).

Biochemistry, hematology, and coagulation tests were assessed in the central laboratory of the Hospital Clínico Universitario of Santiago de Compostela blinded to clinical and neuroimaging data. IL-6, TNFα and sTWEAK measurements were performed in the Clinical Neurosciences Research Laboratory by researchers blinded to clinical and neuroimaging data. Serum levels of IL-6 and sTWEAK were measured by enzyme linked immunosorbent assay (ELISA) technique following manufacturer's instructions. IL-6 ELISA kit (BioLegend, San Diego, USA) minimum assay sensitivity was 1.6 pg/ml with an intra- and inter-assay coefficient of variation (CV) of 5.0 and 6.8%, respectively. sTWEAK Kit (Human TWEAK ELISA Kit (Elabscience, Texas, USA) minimum assay sensitivity was 4.69 pg/mL with an intra- and inter-assay CV of 5.06 and 5.21%, respectively. TNFα was measured using an immunodiagnostic IMMULITE 1000 System (Siemens Healthcare Global, Los Angeles, USA). Minimum assay sensitivity was 1.7 pg/mL, with an inter-assay CV of 6.5% and intra-assay CV of 3.5%. Biomarkers were evaluated within the first 3 months after blood sample collection.

### Endpoints

The main outcome variables were stroke recurrence and the presence and severity of LA evaluated by neuroimaging within the first 48 h after an episode. Secondary endpoints were the association between stroke recurrence and plasma levels of IL-6; TNFα, and sTWEAK.

### Statistical Analysis

For the descriptive study of the quantitative variables we used the mean ± one standard deviation or the median [range] according to the type of distribution determined by the Kolmogorov-Smirnov test for a sample with the significance correction of Lilliefors. The significance of the differences was estimated using the student's *t*-test or the Mann-Whitney U test. One-sided analysis of variance (ANOVA) was used to compare differences between more than two groups. The qualitative variables were expressed as percentages and for the differences the chi-square test and, if applicable, the uncertainty coefficient were used. The estimation of the independent variables associated with stroke recurrence was carried out using multiple regression models, identifying the continuous or categorical variables determined in the first stroke. First, we carried out logistic regression models including all variables with significant differences in univariate studies grouped according to demographic and background data, clinical and progression data and neuroimaging data. With the variables selected, a new logistic regression model was developed, which finally included the results of the biomarker analysis. To detect the ability of biomarkers to classify the values associated with stroke recurrence, ROC (Receiver Operating Characteristic) curves were developed, converting continuous variables into categorical ones for a value that offers maximum sensitivity and specificity. The results were expressed as odds ratio (OR) with 95% confidence intervals (95% CI). Significant values of *p* < 0.05 were considered. Analyzes were performed with IBM SPSS v.25 for Mac.

## Results

**Figure 1:**
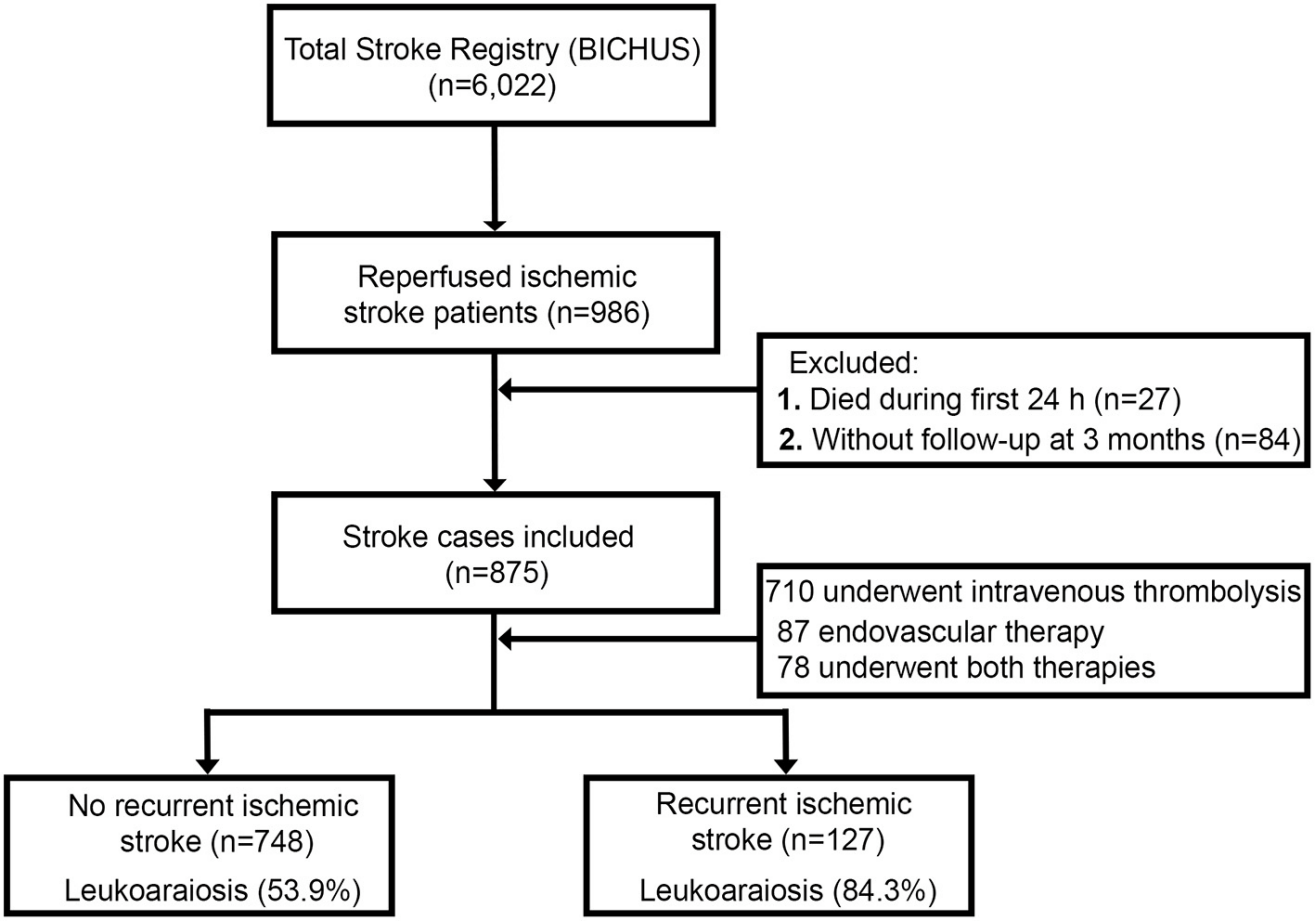
Flowchart of patient groups and LA.

The first patient was enrolled in January 2008 and until the end of the enrollment period (December 2017) 986 reperfused IS patients were registered. Figure 1 lists flowchart of patient groups. We excluded 27 patients who died during the first 24 h and 84 patients for whom no follow-up through either personal interview or IANUS was available. Of the 875 patients eligible for this study (53.9% males; mean age 69.6 ± 11.8 years vs. 46.1% females; mean age 74.9 ± 12.6 years), 710 patients underwent intravenous thrombolysis, 87 endovascular therapy (intraarterial thrombolysis or mechanical thrombectomy) and 78 underwent both intravenous and endovascular therapy. According to the TOAST classification, 206 patients were classified as atherothrombotic (23.5%), 381 as cardioembolic (43.5%), 11 as lacunar (1.3%) and 277 as undetermined (31.7%). Symptomatic hemorrhagic transformation (HT) was noted in 280 (32%) patients during the first admission; of which, 127 suffered stroke recurrence.

The average follow-up time of the study was 25 ± 13 months, during which 127 patients (14.5%) showed stroke recurrence. In the second stroke, 24 patients were classified as atherothrombotic (19.3%), 74 as cardioembolic (59.0%), 25 as undetermined (19.5%) and 3 intracerebral hemorrhages (2.2%). Recurrence was lower in patients with effective reperfusion (4.5 vs. 10.0%, *p* < 0.0001), and in patients undergoing endovascular treatment (6.9%), than in those who received intravenous thrombolysis (14.9%) or those who received both reperfusion therapies (19.2%, *p* < 0.0001). Functional outcome after the second stroke was worse than after the first stroke (mRS at 3 months after the first stroke 1[0, 3] vs. 4 [3, 6] after the second, *p* < 0.0001). Consequently, rates of poor functional outcome (26.1 vs. 85.8%, *p* < 0.0001) and mortality (7.1 vs. 28.3%, *p* < 0.0001) at 3 months were higher after the second stroke.

### Primary Endpoints: The Percentage of Stroke Recurrence and the Severity of LA

**Figure 2:**
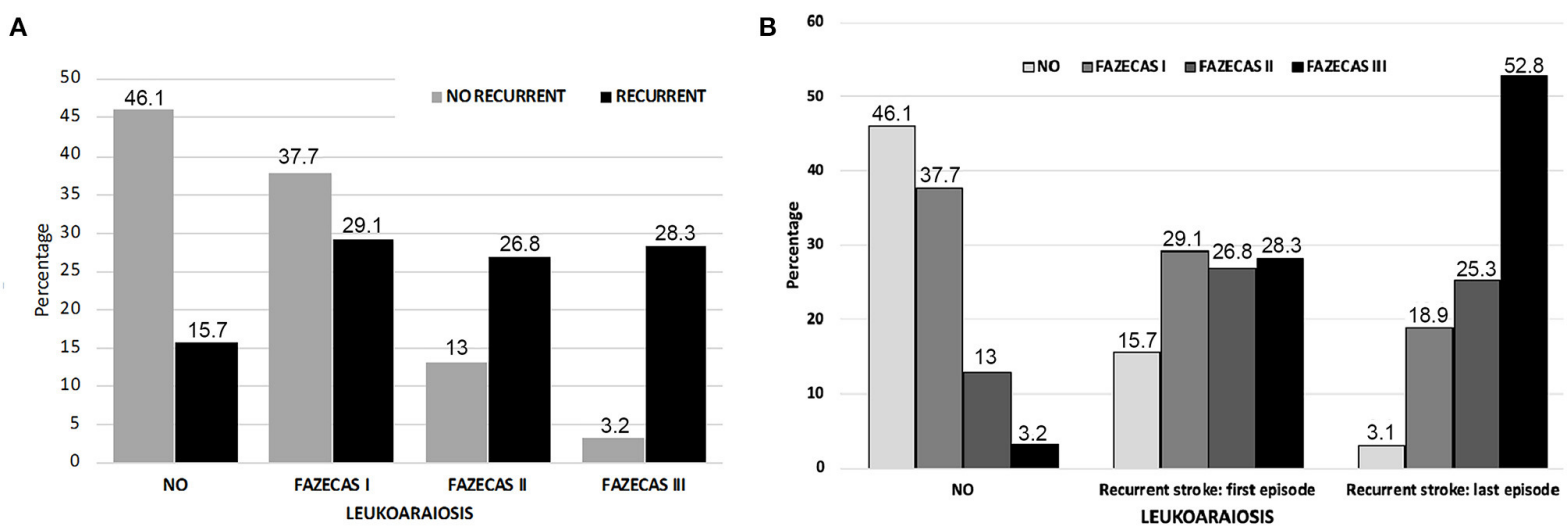
**(A)** Percentage of patients with recurrence according to degree of LA in the initial MRI study. **(B)** Degree of LA in patients who did not present a new stroke; in those who presented a new stroke at the time of the first and in the study conducted following the second stroke.

The univariate analysis of variables obtained at the first admission among patients who did not present with stroke recurrence are expressed in Tables 1, 2. The relationship between the percentage of recurrence (84.2%) and the severity of LA (53.9%) is especially significant (Figure 2A). In the second stroke, the presence of LA was more severe. Grade III of the Fazekas scale went up from 28.3% in the first study to 52.8% in the second (*p* < 0.0001) (Figure 2B). The multivariate model shows that those patients treated with anticoagulant drugs (OR: 3.55; CI 95%: 1.01–6.40; *p* < 0.0001), those with a higher white blood cell count (OR: 1.08; CI 95%: 1.01–1.15; *p* = 0.015) and a greater severity of LA (OR: 23.31; CI 95%: 11.29–48.13; *p* < 0.0001) were independently associated with higher probability of stroke recurrence (Table 3).

**Table 1:** Univariate analysis of demographic variables obtained in the first admission among patients who presented or not a stroke recurrence (*n* = 875).

	**No recurrence** ** (*n*= 748)**	**Recurrence** ** (*n* = 127)**	***p*-value**
**Demographic variables**			
Age, years	71.4 ± 12.7	75.7 ± 10.7	<0.0001
Female gender, %	46.3	44.1	0.700
Arterial hypertension, %	62.0	74.0	0.009
Diabetes, %	22.6	24.4	0.649
Smoking, %	24.5	11.0	0.001
Alcohol consumption, %	4.7	11.2	0.026
Hyperlipidemia, %	38.6	43.3	0.327
Peripheral arterial disease, %	6.3	9.4	0.183
Ischemic heart disease, %	13.1	10.2	0.470
Atrial fibrillation, %	21.1	33.1	0.004
Heart failure, %	4.0	6.3	0.240
Carotid disease, %	0.8	0.8	1.000
Latency time, min^*^	162.1 ± 61.2	160.5 ± 61.5	0.786
Previous antiaggregants, %	25.1	47.2	<0.0001
Previous Anticoagulants, %	6.3	19.7	<0.0001

* *Time between the onset of symptoms and the onset of reperfusion treatment*.

**Table 2:** Univariate analysis of clinical, neuroimaging variables, and molecular markers obtained in the first admission among patients who presented or not a stroke recurrence (*n* = 875).

	**No recurrence** ** (*n* = 748)**	**Recurrence** ** (*n* = 127)**	***p*-value**
**Clinical, Neuroimaging variables**			
Previous mRS	0 [0, 0]	0 [0, 1]	<0.0001
NIHSS at admission	17 (12, 22)	18 (14, 22)	0.041
Early neurological improvement, %	46.3	30.7	0.001
Early neurological deterioration, %	8.5	18.5	0.002
mRS at 3 months	1 [0, 3]	2 [0, 3]	<0.0001
Axillary temperature at admission, °C	36.4 ± 0.7	36.8 ± 0.7	<0.0001
Stroke volume, mL	45.9 ± 70.1	81.0 ± 98.4	<0.0001
Leukoaraiosis, %	53.9	84.3	<0.0001
Leukoaraiosis degree			<0.0001
- No, %	46.1	15.7	
- Grade I, %	37.7	29.1	
- Grade II, %	13.0	26.8	
- Grade III, %	3.2	28.3	
Hemorrhagic transformation			<0.0001
- No, % (patients)	71.1 (532)	49.6 (63)	
- IH1, %	21.5 (161)	20.5 (26)	
- IH2, %	3.9 (29)	13.4 (17)	
- PH1, %	1.9 (14)	9.4 (12)	
- PH2, %	1.6 (12)	7.1 (9)	
TOAST			0.744
- Atherothrombotic, %	23.4	24.4	
- Cardioembolic, %	43.6	43.3	
- Lacunar, %	1.5	–	
- Undetermined, %	31.6	32.3	
**Molecular markers**			
Blood glucose, mg/dl	136.4 ± 53.3	149.3 ± 67.2	0.020
Leukocytes, x10^3^/mL	8.2 ± 3.1	9.6 ± 67.2	0.020
Platelets, x10^3^/mL	204.3 ± 66.8	197.0 ± 69.9	0.257
Fibrinogen, mg/dl	415.3 ± 102.1	438.5 ± 92.6	0.017
C-reactive protein, mg/l	3.9 ± 4.2	4.9 ± 4.6	0.048
Microalbuminuria, mg/24 h	5.3 ± 4.2	7.7 ± 9.4	0.003
LDL-cholesterol, mg/dl	109.6 ± 40.5	98.4 ± 41.0	0.123
HDL-cholesterol, mg/dl	41.7 ± 14.6	41.6 ± 16.7	0.971
Triglycerides, mg/dl	115.4 ± 53.3	102.3 ± 41.0	0.028
Erythrocyte sediment, mm	18.3 ± 20.4	22.3 ± 18.3	0.041

*Modified Rankin scale (mRS); National Institute of Health Stroke Scale (NIHSS)*.

**Table 3:** Logistic regression analysis including anticoagulants, leukocytes, effective reperfusion, and leukoaraiosis degree.

	**Not adjusted**			**Adjusted**		
	**OR**	**CI 95%**	***P*-value**	**OR**	**CI 95%**	***P*-value**
Anticoagulants	3.65	2.16 - 6.20	<0.0001	3.55	1.01 - 6.40	<0.0001
Leukocytes	1.14	1.07 - 1.20	<0.0001	1.08	1.01 - 1.15	0.015
Effective reperfusion	0.51	0.34 - 0.77	0.001	0.97	0.60 - 1.58	0.921
Leukoaraiosis degree						
- Grade I	2.26	1.18–3.99	0.005	2.28	1.28–4.06	0.005
- Grade II	6.05	3.33–10.98	<0.0001	5.11	2.71–9.66	<0.0001
- Grade III	25.87	13.04–51.36	<0.0001	23.31	11.29–48.13	<0.0001

*Dependent variable: Stroke recurrence*.

### Secondary Endpoints: IL-6, TNFα and sTWEAK

**Figure 3:**
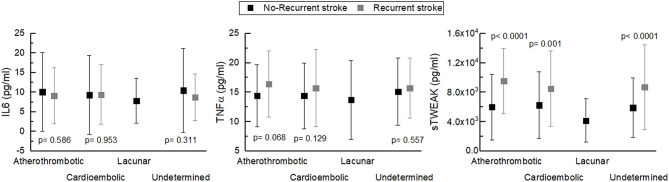
Levels of biomarkers (IL-6, TNFα, and sTWEAK) analyzed in the sample collected during the first stroke, according to the different stroke types, both in those who did not recurred and in those who subsequently recurred.

**Figure 4:**
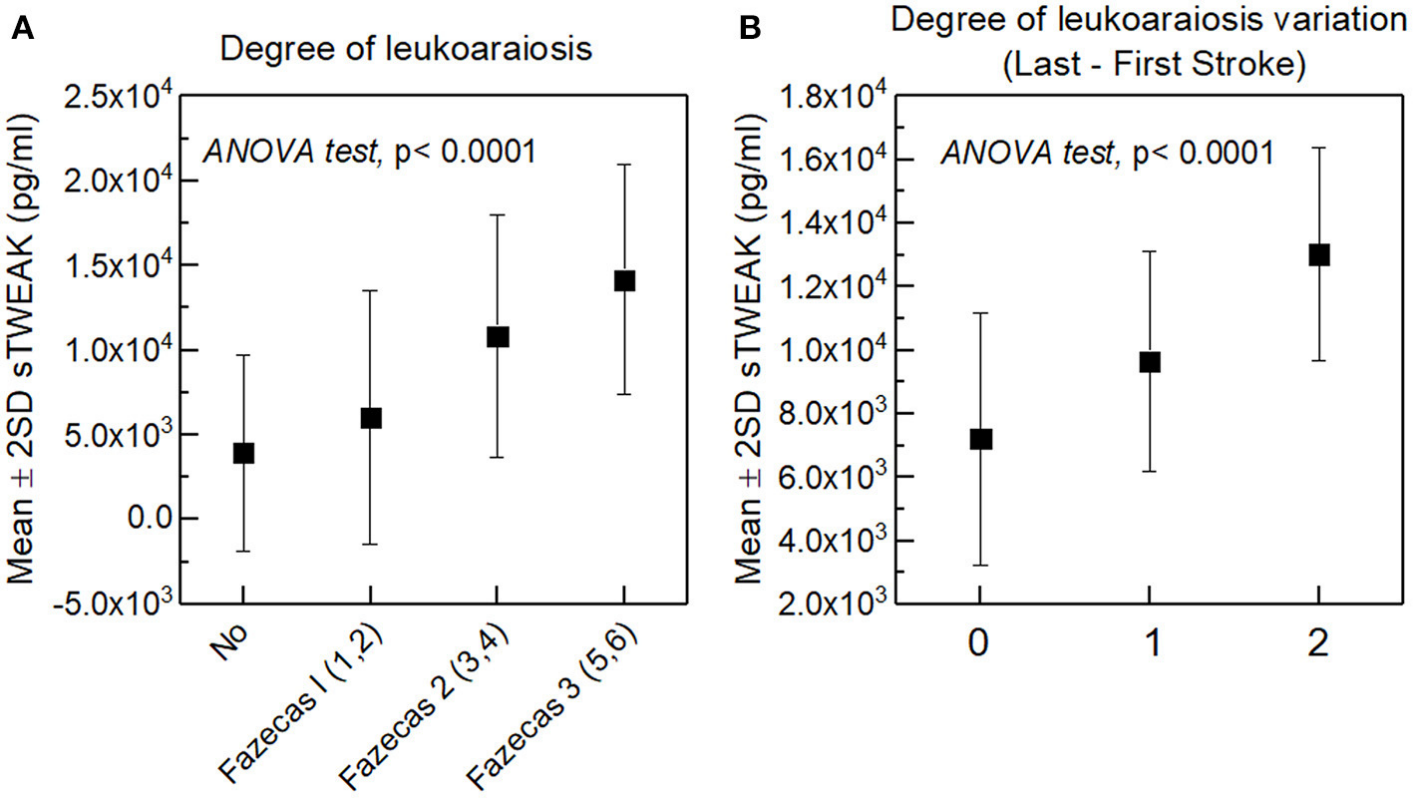
**(A)** Relationship between sTWEAK levels and LA severity at first admission. **(B)** Relationship between sTWEAK levels measured in the last stroke and the variation between the degree of LA found in the last stroke in relation to the first.

IL-6 levels analyzed in the blood sample collected at the first episode at admission and before reperfusion therapies in patients with and without stroke recurrence were similar (9.9 ± 10.4 pg/mL vs. 9.1 ± 7.0 pg/mL, *p* = 0.439), but different for TNFα (14.7 ± 5.6 pg/mL vs. 15.9 ± 5.7 pg/mL, *p* = 0.031) and sTWEAK (5,970.8 ± 4,330.4 pg/mL vs. 8,660.7 ± 5,119.0 pg/mL, *p* < 0.0001). Biomarker levels were similar in different types of stroke, both in patients with and without stroke recurrence. ANOVA tests were performed for IL-6 (*p* = 0.532 vs. *p* = 0.943), for TNFα (*p* = 0.422 vs. *p* = 0.857) and for sTWEAK (*p* = 0.461 vs. *p* = 0.441). sTWEAK levels were higher in all types of recurrent strokes, but similar for IL-6 and TNFα (Figure 3). In recurrent strokes, biomarker measurements were similar in the sample collected in the first and in the second episode (IL-6, 9.1 ± 6.9 pg/mL vs. 9.4 ± 7.5 pg/mL, *p* = 0.330; TNFα, 15.8 ± 5.8 pg/mL vs. 16.0 ± 7.0 pg/mL, *p* = 0.168; sTWEAK, 8,763.4 ± 5,167.3 pg/mL vs. 8,767.3 ± 4,126.0 pg/mL, *p* = 0.992). We demonstrated a correlation between sTWEAK levels and the severity of LA at the first admission (Spearman's coefficient *p* < 0.0001) (Figure 4A) that does not exist with the other biomarkers, and that the levels of sTWEAK measured in the second episode at admission increased in those patients in whom the severity of LA progressed between the two or more episodes as shown in Figure 4B (Spearman's coefficient *p* < 0.0001).

The ROC curve analysis of sTWEAK for stroke recurrence shows an area under the curve of 0.651; CI 95%: 0.596–0.705; *p* < 0.0001. For a cut-off point of 7,000 pg/mL, sensitivity is 63% and specificity 64%. In a logistic regression model adjusted for all biomarkers, only the sTWEAK values ≥7,000 pg/mL measured in the first stroke were independently associated with stroke recurrence (OR: 2.79; CI 95%: 1.87–4.16; *p* < 0.0001).

When the sTWEAK categorized variable was introduced into the logistic regression model, but not LA, sTWEAK multiplied the risk of recurrence by 2.48 (Table 4, Model A). It was demonstrated that if we include LA as a simple categorical variable, levels of sTWEAK, measured at the onset of the first stroke, ≥7,000 pg/mL multiplies by 1.62 the risk of presenting a recurrent stroke (Table 4, Model B). Importantly, however, if we include the different degrees of LA severity, the value of sTWEAK ≥7,000 pg/mL as a predictor of recurrence risk is no longer independent and is subrogated to LA severity (Table 4, Model C).

**Table 4:** Multivariate analysis including biomarkers, leukoaraiosis and leukoaraiosis degree. Dependent variable: Recurrence.

	**Not adjusted**			**Adjusted**		
	**OR**	**CI 95%**	**p value**	**OR**	**CI 95%**	**p value**
**MODEL A**						
Anticoagulants	3.65	2.16 - 6.20	<0.0001	3.64	2.09 - 6.31	<0.0001
Leukocytes	1.14	1.07 - 1.20	<0.0001	1.11	1.05 - 1.17	<0.0001
Effective reperfusion	0.51	0.34 - 0.77	0.001	0.64	0.42 - 0.98	0.041
sTWEAK ≥ 7,000 pg/mL	2.89	1.96 - 4.27	<0.0001	2.48	1.65 - 3.72	<0.0001
**MODEL B**						
Anticoagulants	3.65	2.16 - 6.20	<0.0001	3.64	2.08 - 6.41	<0.0001
Leukocytes	1.14	1.07 - 1.20	<0.0001	1.10	1.04 - 1.17	0.001
Effective reperfusion	0.51	0.34 - 0.77	0.001	0.68	0.44 - 1.05	0.083
Leukoaraiosis	4.58	2.78 - 7.54	<0.0001	3.34	1.94 - 5.76	<0.0001
sTWEAK ≥ 7,000 pg/mL	2.89	1.96 - 4.27	<0.0001	1.62	1.04 - 2.53	0.032
**MODEL C**						
Anticoagulants	3.65	2.16 - 6.20	<0.0001	3.52	1.96 - 6.34	<0.0001
Leukocytes	1.14	1.07 - 1.20	<0.0001	1.08	1.02 - 1.15	0.014
Effective reperfusion	0.51	0.34 - 0.77	0.001	0.96	0.59 - 1.56	0.872
Leukoaraiosis degree						
- Grade I	2.26	1.18 - 3.99	0.005	2.42	1.34 - 4.33	0.003
- Grade II	6.05	3.33 - 10.98	<0.0001	6.20	2.91 - 13.17	<0.0001
- Grade III	25.87	13.04 - 51.36	<0.0001	29.33	12.25 - 69.75	<0.0001
sTWEAK ≥ 7,000 pg/mL	2.89	1.96 - 4.27	<0.0001	0.76	0.43 - 1.35	0.350

## Discussion

Stroke recurrence is the first cause of increased mortality and non-motor sequelae, and this complication persists in patients undergoing reperfusion treatments (1, 5–9, 12–14, 28). In our series of patients with acute IS, who received the best possible treatment according to management guidelines, recurrence was 14.5% for an average follow-up time of 2 years. The outcome of patients with recurrence was poor in 86% of cases, with a mortality of 28%. Recurrence in our study is similar to that obtained by several authors (29), but higher than that referred in other studies. This may be explained by the fact that our follow-up time was longer, and the age of our patients was higher. Mortality, however, was similar in all the studies reviewed (6, 12, 30, 31).

The type of stroke did not influence the frequency of recurrence, although the second episode led to the reclassification of almost 50% of the undetermined strokes into cardioembolic, and three patients with cardioembolic strokes recurred as intracerebral hemorrhage. Recurrence has been significantly lower in patients undergoing thrombectomy than in the case of systemic thrombolysis, and much lower than when the procedure was combined. Previously published data are uneven (1, 12–14, 32, 33). In our cases, these results were not influenced by the time between the onset of symptoms and treatment (*p* = 0.108), or follow-up time (*p* = 0.424, data not shown). However, patients in whom an effective reperfusion was achieved presented with lower recurrence rates. In a previous research work, we found that the treatment with tPA without reperfusion is associated with a worse patient progression, possibly due to the toxic effect of the drug in these cases (34).

In our study, oral anticoagulation and white blood cell count in the first stroke were independent factors associated with stroke recurrence. Although in the first episode the frequency of cardioembolic subtype was similar in both groups, in the second episode half of undetermined strokes were reclassified into cardioembolic, which implies an undervaluation of the initial diagnosis of cardioembolic. The platelet count was similar in both groups and functional situation before stroke was worse in the patients who recurred, although this data did not reach independence in the multivariate model (13).

It is interesting that LA has been the strongest factor associated with stroke recurrence, and this association is directly related to the severity and extent of the white matter lesion. Despite the differences in the neuroimaging study and the method to quantify LA, this association is widely reported in the literature (35–41). There are, however, some differential data: (1) the association with lacunar infarctions (35) (in our series, only in reperfused patients, and none in the 11 lacunar infarctions recurred), and (2) the relationship with cardioembolic infarctions, which is not found in any study (38, 39). In our case, the association between LA and recurrence was similar in atherothrombotic, cardioembolic and undetermined strokes (*p* = 0.383). A possible explanation for this discrepancy might be that in our series the patients with cardioembolic strokes had a more advanced age (atherothrombotic 69.6 ± 12.6 years, cardioembolic 73.8 ± 11.8 years, lacunar 67.3 ± 11.8 years and undetermined 71.8 ± 12.9 years). Aside from theses discrepancies, LA is currently an important factor of poor outcome after a stroke.

Of the determined inflammatory markers (white blood cells, fibrinogen, C-reactive protein, sedimentation rate, IL-6 and TNFα), only white blood cells and TNFα maintained statistical significance in the first regression models but they lost it when including all clinical factors and of neuroimaging. However, sTWEAK (levels ≥ 7,000 pg/mL) was associated independently with an increased risk of stroke recurrence. The strong relationship between the sTWEAK levels in the first stroke and the severity of LA suggests that sTWEAK is a surrogate marker for LA, and thus, when we included the severity of LA in the regression model, sTWEAK disappeared as an independent recurrence factor (Table 4, Model B).

sTWEAK is a type II transmembrane glycoprotein of the TNF (tumor necrosis factor) superfamily that acts by binding to Fn14 which is a small transmembrane type I protein. TWEAK-Fn14 is expressed in all cells that act in the Neurovascular Unit and overexpresses within a few hours of establishing a cerebral ischemia (42–45). TWEAK-Fn14 overexpression induces an inflammatory profile in brain endothelial cells with increased secretion of proinflammatory cytokines, production and activation of matrix metalloproteinases that will participate in the disruption of the blood-brain barrier and expression of intercellular adhesion molecules involved in the union of white blood cells to the endothelium (46, 47). This maintained expression could condition the development and progression of LA and could be the molecular marker associated with white matter disease associated with chronic cerebral ischemia. This hypothesis, however, remains to be demonstrated.

From a clinical point of view, the importance of sTWEAK as predictor of LA progression associated with the increase of stroke recurrence does not seem preferred, since neuroimaging is more sensitive and specific, at least with the method used (we have exclusively determined sTWEAK, and no sTWEAK-Fn14). However, the possibility of blocking the activation of the sTWEAK-Fn14 system (anti-sTWEAK or anti-Fn14 monoclonal antibodies, or through sTWEAK-Fn14 fusion blockade) makes this marker a hopeful therapeutic target that could decrease the progression of LA and stroke recurrence (48, 49).

This study has some limitations. First, our study presents the weaknesses of any retrospective study, even if its origin is prospective. Bias in the enrolment of patients was reduced as we enrolled all those registered in our hospital and followed-up in any hospital of the public system (in Galicia, the network of private hospitals is small). Second, sTWEAK measurements were not simultaneous and were made by different researchers, although measurements were always blind to the clinical and neuroradiological data and supervised by the same senior researchers, and the same is true of clinical and neuroradiological data. Three, it is important to note that LA is a gradual disease affected by different risk factors, and not associated with a unique pathological process (16, 50). There is the possibility that LA may be associated with factors in the study population other than stroke. It is known that in regions corresponding to LA on neuroimaging, the wall of penetrating arteries is thickened and hyalinized, and there is often narrowing, elongation, and tortuosity of small vessels, potentially leading to reduced cerebral blood flow, and permanent BBB damage. Furthermore, after the first stroke, Wallerian degeneration (WD) could develop and cause new white matter hyperintensities related with LA progression (51). Four, serum levels of sTWEAK do not represent a specific marker of a particular process; patients with multiple sclerosis, heart failure, or atherosclerosis show also variations in the sTWEAK levels (52). However, we investigate the possible relationship among sTWEAK—LA—stroke recurrence in reperfused IS patients. The strong points of this work are the unbiased screening of individuals, the high number of enrolled patients, and the large number of biomarkers assessed.

## Conclusion

Stroke recurrence is associated with increased mortality, non-motor sequelae. Currently, preventive efficacy is limited. The presence of an advanced degree of LA, as well as its progression, is the main neuroimaging factor associated with stroke recurrence. sTWEAK (≥ 7,000 pg/mL) is a biomarker correlated with the progression of LA and stroke recurrence. sTWEAK could become a diagnostic boimarker and a potential therapeutic target in reducing stroke recurrence but further studies will be necessary.

## Data Availability Statement

The original contributions generated for this study are included in the article/supplementary material, further inquiries can be directed to the corresponding author/s.

## Ethics Statement

The studies involving human participants were reviewed and approved by This research was carried out in accordance with the Declaration of Helsinki of the World Medical Association (2008) and approved by the Ethics Committee of Santiago de Compostela (2019/616). The patients/participants provided their written informed consent to participate in this study.

## Author Contributions

PH, RI-R, and JC: conception and design of the study. FC, AdS-C, TS, and MP-M: data acquisition and analysis. JC, MR-Y, JP, and IL-D: clinical data acquisition and analysis. RI-R, TS, JC, PH, and AdS-C: handled funding and supervision. PH and JC: statistical analysis. RI-R, PH, and JC: manuscript drafting. MP-M, TS, JC, and FC: critical revision for important intellectual content. JC, MR-Y, JP, IL-D: supervision. All authors reviewed and approved the manuscript.

## Conflict of Interest

The authors declare that the research was conducted in the absence of any commercial or financial relationships that could be construed as a potential conflict of interest.
